# Subterranean morphology underpins the degree of mycoheterotrophy, mycorrhizal associations, and plant vigor in a green orchid *Oreorchis patens*

**DOI:** 10.1111/tpj.70045

**Published:** 2025-02-19

**Authors:** Kenji Suetsugu, Hidehito Okada

**Affiliations:** ^1^ Department of Biology, Graduate School of Science Kobe University Kobe Japan; ^2^ Institute for Advanced Research Kobe University Kobe Japan

**Keywords:** Calypsoinae, mixotrophy, mycorrhizas, Orchidaceae, partial mycoheterotrophy, phenotypic plasticity, saprotrophic fungi, stable isotopes

## Abstract

The evolution of full heterotrophy is a fascinating topic in plant evolution, with recent studies suggesting that partial mycoheterotrophy (mixotrophy) serves as a transitional stage toward full mycoheterotrophy in orchids. However, the adaptive significance of fungal‐derived carbon in mixotrophic plants remains largely unexplored. In this study, we investigated the photosynthetic orchid *Oreorchis patens*, a species related to the leafless genus *Corallorhiza* within the subtribe Calypsoinae. Using high‐throughput DNA sequencing, ^13^C and ^15^N isotopic analyses, and phenotypic evaluations, we explored the role of coralloid rhizomes – a feature common in fully mycoheterotrophic orchids – in fungal partnerships, the degree of mycoheterotrophy, and plant vigor. Our findings reveal that *O. patens* plants with coralloid rhizomes predominantly associate with saprotrophic Psathyrellaceae fungi, whereas those without coralloid rhizomes also partner with rhizoctonias and other potentially orchid mycorrhizal fungi. Notably, plants with coralloid rhizomes exhibited enriched ^13^C signatures, indicating a greater reliance on fungal‐derived carbon. These plants also demonstrated more vigorous flowering scapes and produced a higher number of flowers, suggesting that mycoheterotrophy significantly enhances plant vigor. This study provides rare insights into the adaptive significance of mycoheterotrophy. Recent research suggests that some partially mycoheterotrophic orchids can adjust their heterotrophic status to optimize carbon resource use under specific conditions, such as low‐light environments. However, an increased proportion of fungal‐derived carbon may sometimes merely reflect reduced photosynthesis in such conditions, thereby amplifying the apparent contribution of fungal‐derived carbon. Our findings offer more direct evidence that carbon acquisition via mycoheterotrophy is beneficial for partially mycoheterotrophic orchids.

## Significance Statement

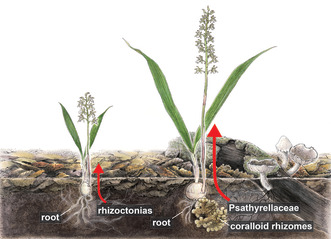

The evolution of full heterotrophy from photosynthetic ancestors is a longstanding question in evolutionary ecology. Our study demonstrates that coralloid rhizomes in a photosynthetic orchid promote greater mycoheterotrophic carbon acquisition, leading to more vigorous flowering scapes and increased flower production. These findings not only provide evidence that mycoheterotrophy enhances plant fitness but also offer insights into why, under specific environmental conditions, certain plants shift their carbon acquisition strategies toward heterotrophy.

## INTRODUCTION

In typical mycorrhizal symbiosis, fungi receive photosynthetically derived carbon (C) from plants while providing essential inorganic, and sometimes organic, nutrients such as nitrogen and phosphate, and water from the soil (Heijden et al., 2015; Smith & Read, 2008). Mycoheterotrophic plants, however, deviate from this norm; these non‐photosynthetic plants rely entirely on mycorrhizal fungi for mineral and carbon nutrition without offering any discernible advantage to their mycobionts (Selosse & Rousset, 2011).

The evolution of fully mycoheterotrophic plants is one of the most intriguing topics in plant evolution (Leake, 1994; Merckx, 2013; Shefferson et al., 2024). Orchidaceae, with approximately 28 000 species, provides a prime setting to study this phenomenon, as full mycoheterotrophy has independently emerged at least 30 times within the family (Merckx & Freudenstein, 2010). All orchids are initially mycoheterotrophic due to their lack of endosperm and sufficient carbon reserves (Leake, 1994; Merckx, 2013). This initial strategy may still align with mutualistic symbiosis, as fungi eventually receive carbon from mature plants – a concept termed the “take now, pay later” hypothesis (Cameron et al., 2008; Field, Leake, et al., 2015; Field, Pressel, et al., 2015; Read et al., 2024).

However, numerous photosynthetic plants initially exhibit mycoheterotrophic habits and maintain a nutritional strategy combining autotrophy and mycoheterotrophy during adulthood (partial mycoheterotrophy or mixotrophy) (Bidartondo et al., 2004; Gebauer & Meyer, 2003; Hynson et al., 2013; Selosse & Roy, 2009). Partial mycoheterotrophy was first suspected in orchids of the genera *Cephalanthera* and *Epipactis* (Neottieae), based on albino mutants of typically green taxa (Julou et al., 2005; Selosse et al., 2004). More robust evidence came from the natural abundances of ^13^C and ^15^N in these plants, which are intermediate between autotrophic and fully mycoheterotrophic plants (Bidartondo et al., 2004; Gebauer & Meyer, 2003; Julou et al., 2005). Subsequent studies have reported partial mycoheterotrophy in other orchids across various tribes and in other green plants (Bidartondo et al., 2004; Gebauer et al., 2016; Giesemann et al., 2020; Motomura et al., 2010; Suetsugu, Haraguchi, et al., 2021; Suetsugu & Matsubayashi, 2021a; Suetsugu, Taketomi, et al., 2020; Tedersoo et al., 2007).

Partial mycoheterotrophy is often considered a precursor to full mycoheterotrophy (Jacquemyn & Merckx, 2019; Selosse & Roy, 2009). Phylogenetic evidence suggests that partial mycoheterotrophy likely emerged before full mycoheterotrophy, potentially facilitating its later development (Jacquemyn & Merckx, 2019; Motomura et al., 2010; Selosse & Roy, 2009). Therefore, partially mycoheterotrophic species likely represent an evolutionary bridge leading to full mycoheterotrophy. Mycoheterotrophic evolution in orchids often involves shifts in mycorrhizal fungal partners likely to accommodate the increased organic carbon demands (Bidartondo et al., 2004; Ogura‐Tsujita et al., 2012; Wang et al., 2021; Yagame et al., 2016). While most green‐leaved orchids form associations with non‐ectomycorrhizal (ECM) rhizoctonias, including Ceratobasidiaceae, Tulasnellaceae, and Serendipitaceae (Dearnaley et al., 2012; Rasmussen & Rasmussen, 2014), fully mycoheterotrophic orchids and partially mycoheterotrophic orchids with pronounced heterotrophy recruit ECM or saprotrophic non‐rhizoctonia fungi as their mycobionts (Bidartondo et al., 2004; Hynson et al., 2013; Jacquemyn & Merckx, 2019; Ogura‐Tsujita et al., 2009; Suetsugu et al., 2022; Suetsugu & Matsubayashi, 2021b; Taylor & Bruns, 1997). Intriguingly, some fully mycoheterotrophic plants even shift fungal partnerships during ontogeny to support the greater carbon demands of adult plants compared to underground seedling (protocorm) development (e.g., from litter‐decaying *Mycena* to wood‐decaying *Armillaria* fungi) (Xu & Guo, 1989; Xu & Mu, 1990).

The genus *Oreorchis*, part of the subtribe Calypsoinae (Epidendreae, Epidendroideae), includes approximately 19 species distributed from the Himalayas to Taiwan, eastern Siberia, Korea, and Japan (Chase et al., 2015; Li et al., 2020; Yang et al., 2021). *Oreorchis* is phylogenetically closely related to the leafless genus *Corallorhiza*, suggesting potential mycoheterotrophic tendencies, given that partially mycoheterotrophic orchids are often closely related to fully mycoheterotrophic counterparts (Jacquemyn & Merckx, 2019; Selosse & Roy, 2009). *Oreorchis indica* exhibits a relatively high degree of mycoheterotrophy, obtaining approximately 40% of its carbon from ECM *Tomentella* (Suetsugu, Haraguchi, et al., 2021). This suggests that the shift to full mycoheterotrophy within the *Oreorchis*–*Corallorhiza* lineage was likely facilitated through partial mycoheterotrophy associated with the recruitment of an ECM fungus.

**Figure 1:**
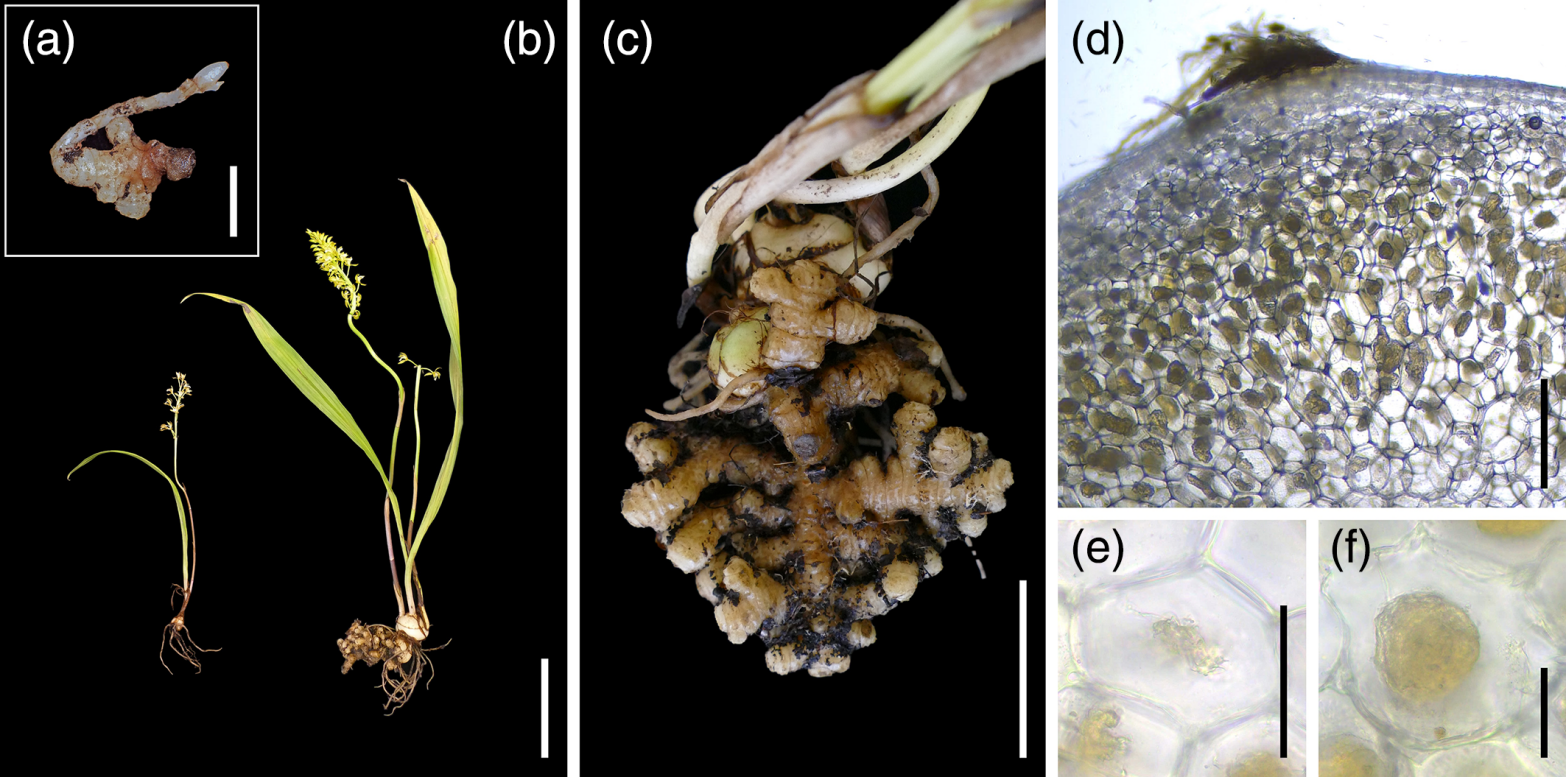
*Oreorchis patens* and its mycorrhizal interaction. (a) Protocorm. (b) Comparison between a plant with a coralloid rhizome and one without a coralloid rhizome. (c) Coralloid rhizome. (d) Cross section of a coralloid rhizome. (e) Close‐up of a coralloid rhizome cortical cell with undegenerated fungal coils. (f) Close‐up of a coralloid rhizome cortical cell with degenerated fungal coils. Scale bars: 5 mm, 10 cm (b), 5 cm (c), 1 mm (d), and 200 μm (e, f).

Meanwhile, both plastome and nuclear ITS phylogenetic analyses indicate that *Oreorchis* is likely not monophyletic, comprising two distinct groups: one represented by *O. indica* and the other by *O. patens* (Barrett et al., 2025; Freudenstein et al., 2017; Kim et al., 2020; Li et al., 2020; Yang et al., 2021). Specifically, these analyses generally support that *O. patens*, *O. coreana*, and *O. fargesii* form an early‐diverging group that is sister to *O. indica*, *O. erythrochrysea*, and *Corallorhiza* (Barrett et al., 2025; Freudenstein et al., 2017; Kim et al., 2020; Li et al., 2020; Yang et al., 2021). Thus, the potential mixotrophy in *O. patens* warrants further investigation. Notably, *O. indica* often displays multi‐branched coralloid rhizomes, a characteristic of mycoheterotrophic plants (Leake, 1994; Merckx, 2013), while *O. patens* infrequently shows such structures in mature stages (Figure 1), suggesting it may be less suited for a partially mycoheterotrophic strategy.

Interestingly, rare mature *O. patens* plants with coralloid rhizomes were observed solely near decomposed fallen trees, hinting at mixotrophy with wood‐decomposing fungi. This contrasts with *Corallorhiza* and *O. indica*, which associate with ECM fungi (Barrett et al., 2010, 2022; Suetsugu, Haraguchi, et al., 2021; Taylor et al., 2004; Taylor & Bruns, 1997, 1999; Zimmer et al., 2008). Meanwhile, historical observations report coralloid rhizomes in the green Calypsoinae orchid *Aplectrum hymenale* near decaying wood (Gillman, 1876; MacDougal, 1899; MacDougal & Dufrenoy, 1944). Considering these anecdotal reports and the recruitment of wood‐decaying fungi likely facilitating photosynthesis loss in the *Cremastra appendiculata* species complex, another Calypsoinae member (Suetsugu et al., 2022), associations with wood‐decaying fungi might have promoted higher heterotrophy in *O. patens*.

So far, the adaptive significance of heterotrophic carbon gain in plants remains incompletely understood (Roy et al., 2013; Selosse et al., 2017). Several studies suggest that partial heterotrophy is a flexible mechanism for efficient carbon use and could be an adaptive strategy (Jacquemyn & Merckx, 2019). For instance, two partially mycoheterotrophic *Cephalanthera* species greatly supplement their carbon supply from fungal partners under low‐light conditions but become nearly autotrophic under sufficient light (Preiss et al., 2010). Additionally, artificial shading increases the proportion of host‐derived carbon in hemiparasitic *Rhinanthus* species (Těšitel et al., 2011). These studies suggest context‐dependent fungal‐derived carbon usage in mixotrophic plants. However, the increased ratio of host‐derived carbon might reflect reduced photosynthesis under low light, which concentrates the contribution of host‐derived carbon. Further research is needed to determine whether increased heterotrophy provides actual benefits.

Here, we investigated the fungal partners of *O. patens* plants with and without coralloid rhizomes to explore whether they exploit ECM (as with their congeners) or saprotrophic non‐rhizoctonia fungi (as inferred from environmental circumstantial evidence) through high‐throughput DNA sequencing. Additionally, we assessed the abundance of their ^13^C and ^15^N stable isotopes to evaluate the hypothesis that the subterranean morphology of *O. patens* specimens is linked to their mycorrhizal communities and nutritional mode. Finally, we investigated whether *O. patens* specimens with coralloid rhizomes exhibit taller flowering scapes with a greater abundance of flowers (as a proxy for vigor).

## RESULTS

### Impact of coralloid rhizomes on other traits

*Oreorchis patens* plants with coralloid rhizomes exhibited significantly longer flowering scapes (364.2 ± 84.9 mm, *n* = 9; *P* = 0.04) compared to those without coralloid rhizomes (270.0 ± 67.3 mm, *n* = 6). Additionally, these plants produced a significantly higher number of flowers (34.2 ± 10.1, *n* = 9; *P* = 0.04) than their counterparts without coralloid rhizomes (23.3 ± 7.4, *n* = 6). The trend extended to leaf dimensions; *O. patens* plants with coralloid rhizomes generally had longer and wider leaves, although the differences were not statistically significant (317.3 ± 79.7 mm in length and 25.2 ± 4.4 mm in width, *n* = 9, compared to 252.4 ± 79.9 mm in length and 21.9 ± 5.5 mm in width, *n* = 10; *P* = 0.09 in length and *P* = 0.17 in width). There was no significant difference in the number of flowering scapes (*P* = 0.18) and leaves (*P* = 0.20) per individual between the two groups. Simultaneously, both groups exhibited *F*_v_/*F*_m_ values within the typical range of 0.7–0.83 for healthy autotrophic plants (Maxwell & Johnson, 2000; Ritchie, 2006). No significant differences were observed between *O. patens* with coralloid rhizomes (0.750 ± 0.021, *n* = 9) and those without coralloid rhizomes (0.753 ± 0.022, *n* = 10; *P* = 0.77).

### Molecular identification of mycobionts

**Figure 2:**
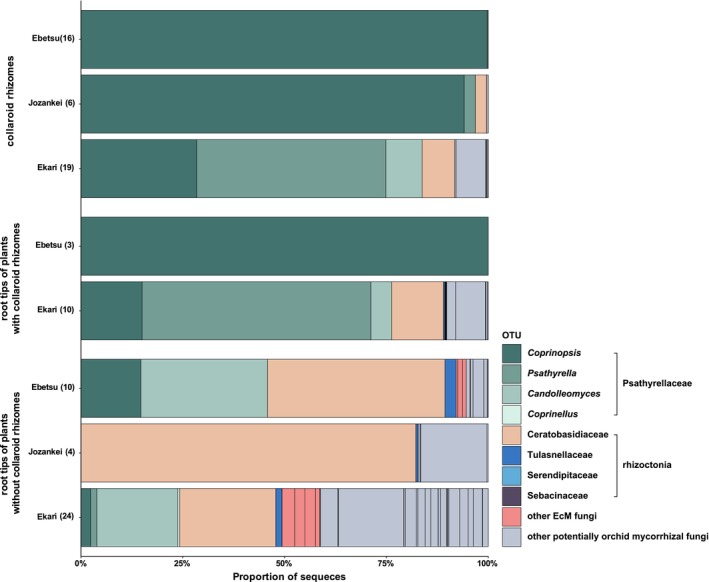
Relative abundance of mycorrhizal communities associated with *Oreorchis patens*. The numbers following the locality names indicate the number of specimens examined.

**Figure 3:**
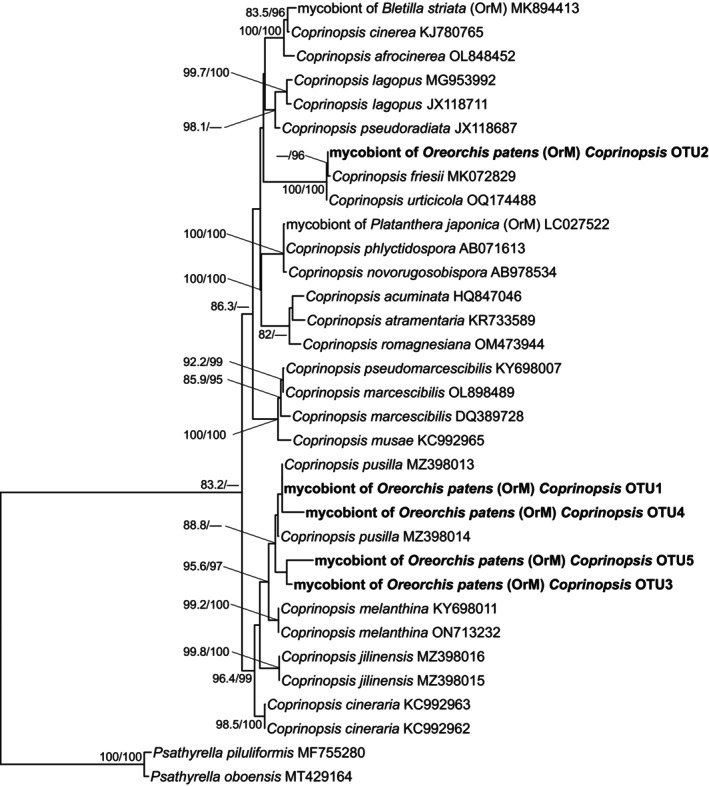
Phylogenetic tree of ITS2 rDNA sequences from *Coprinopsis* OTUs detected in mycorrhizal samples of *Oreorchis patens* (in bold), along with sequences obtained from the INSDC database. The OTUs detected in *O. patens* are ranked by the number of sequencing reads. Accession numbers are provided for all INSDC sequences. The tree is rooted using *Psathyrella piluliformis* and *Psathyrella oboensis* (Psathyrellaceae). Nodes with SH‐aLRT values <80% and ultrafast bootstrap values <95% are not shown. Scale bar indicates the number of substitutions per site. OrM, orchid mycorrhizal fungi; OTU, operational taxonomic unit.

Community profiling using metabarcoding revealed that *O. patens* with coralloid rhizomes predominantly associates with fungi in the family Psathyrellaceae across all three populations examined (10 operational taxonomic units [OTUs], 353 963 reads, accounting for 90.98% of all reads; Figure 2; Table S1). Psathyrellaceae taxa were overwhelmingly dominant in the coralloid rhizomes of coralloid rhizome‐bearing individuals (324 407 reads, 92.17%), while Psathyrellaceae also remained the primary mycorrhizal symbionts in their roots (52 676 reads, 85.14%) (Figure 3). However, as suggested in previous studies on Calypsoinae orchids (Suetsugu et al., 2022; Yagame et al., 2013), the primary mycorrhizal organs of coralloid rhizome‐bearing individuals appear to have shifted from roots to coralloid rhizomes. Therefore, the roots of coralloid rhizome‐bearing individuals exhibited extremely low rates of mycorrhizal colonization. Notably, in the Jozankei population, no mycorrhizal roots were found in coralloid rhizome‐bearing individuals.

Among the identified mycobionts, *Coprinopsis* spp. (four OTUs, 263 820 reads, 63.74% of all reads in samples with coralloid rhizomes) were the main fungal partners of *O. patens* with coralloid rhizomes. In contrast, OTUs from typical orchid mycorrhizal families – Ceratobasidiaceae (seven OTUs, 19 174 reads, 4.63%), Tulasnellaceae (three OTUs, 103 reads, 0.025%), and Serendipitaceae (two OTUs, 67 reads, 0.016%) – were detected at much lower levels in these samples.

On the other hand, *O. patens* specimens without coralloid rhizomes showed more frequent associations with typical orchid mycorrhizal families. Specifically, Ceratobasidiaceae OTUs (seven OTUs, 72 182 reads, 45.78% of all reads in samples without coralloid rhizomes) were the most abundant fungal partners (Figure 2). Psathyrellaceae OTUs were the second most dominant mycobionts in these specimens (13 OTUs, 45 054 reads, 28.58% of all reads), although their association was less prevalent compared to individuals with coralloid rhizomes.

**Figure 4:**
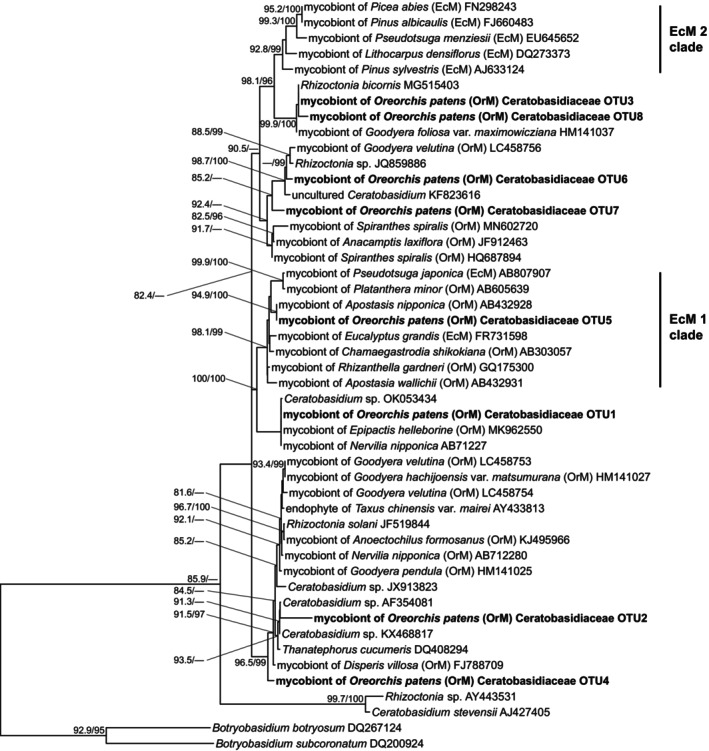
Phylogenetic tree of ITS2 rDNA sequences from Ceratobasidiaceae OTUs detected in mycorrhizal samples of *Oreorchis patens* (in bold), along with sequences obtained from the INSDC database. The OTUs detected in *O. patens* are ranked by the number of sequencing reads. Accession numbers are provided for all INSDC sequences. The tree is rooted using *Botryobasidium botryosum* and *Botryobasidium subcoronatum* (Botryobasidiaceae). Nodes with SH‐aLRT values <80% and ultrafast bootstrap values <95% are not shown. Scale bar indicates the number of substitutions per site. EcM, ectomycorrhizal fungi; OrM, orchid mycorrhizal fungi; OTU, operational taxonomic unit.

Molecular phylogenetic analysis revealed that the most abundant fungus (*Coprinopsis* OTU1; 257 951 reads, 62.33% of all reads in samples with coralloid rhizomes) in *O. patens* with coralloid rhizomes is closely related to *Coprinopsis pusilla* (Figure 3). Most *Coprinopsis* OTUs, including *Coprinopsis* OTU1, have not been reported as mycobionts in orchids (Figure 3; Figures S1–S3). However, other Psathyrellaceae OTUs (e.g., *Psathyrella*, *Coprinellus*, and *Candolleomyces*) were closely related to mycobionts previously identified in orchids such as *Cremastra variabilis*. Additionally, the phylogenetic analysis indicated that most Ceratobasidiaceae and Tulasnellaceae OTUs are related to mycobionts found in other rhizoctonia‐associated orchids (Figure 4; Figures S4 and S5). Meanwhile, Ceratobasidiaceae OTU6, a minor mycobiont for both *O. patens* with (631 reads) and without coralloid rhizomes (82 reads), belongs to the ECM‐forming clade within Ceratobasidiaceae (Veldre et al., 2013).

### Stable isotope analysis

**Figure 5:**
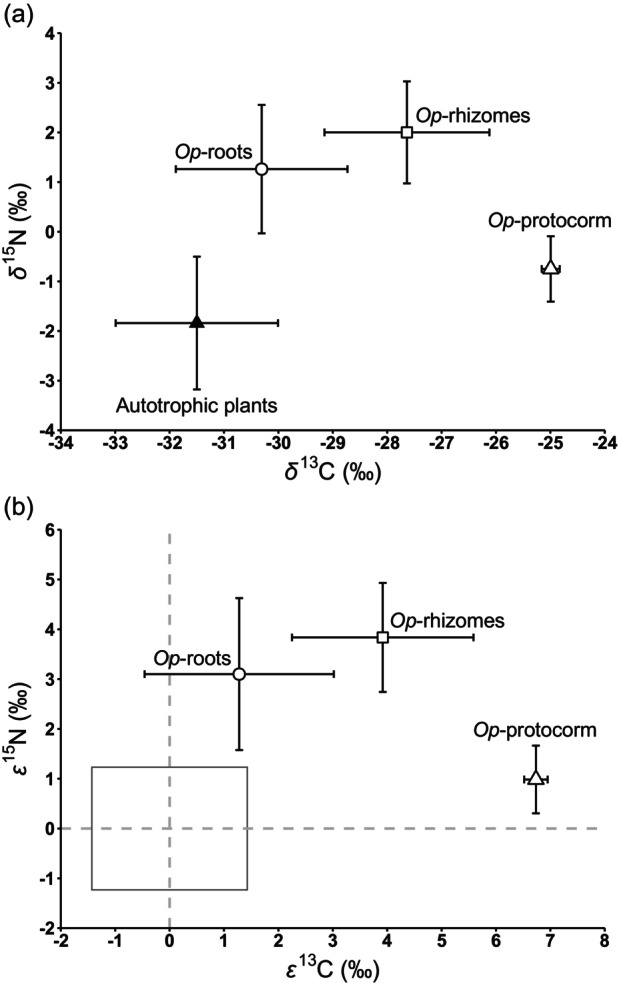
Mean (±SD) values of δ^13^C and δ^15^N (a) or ε^13^C and ε^15^N (b) in *Oreorchis patens* and its neighboring autotrophic plants. *Oreorchs patens* is categorized into three groups: specimens with coralloid rhizomes (*Op*‐rhizomes), without coralloid rhizomes (*Op*‐roots), and protocorms (*Op*‐protocorm). The green box represents the mean enrichment factors (±1 SD) of autotrophic plants.

The protocorm stage of *O. patens* exhibited significant ^13^C enrichment compared to autotrophic reference plants, with a ^13^C enrichment factor of 6.7 ± 0.2‰ (*n* = 5, *P* < 1.0 × 10^−7^). Additionally, *O. patens* samples with coralloid rhizomes displayed a ^13^C isotopic signature intermediate between autotrophic plants and the protocorm stage (Figure 5; Table S2). The δ^13^C values for these samples (−27.6 ± 1.5‰, *n* = 15) were significantly higher than those of autotrophic reference plants (−31.5 ± 1.5‰; *n* = 59, *P* < 1.0 × 10^−7^) and *O. patens* samples without coralloid rhizomes (−30.3 ± 2.0‰, *n* = 18; *P* < 1.0 × 10^−7^).

The δ^15^N values showed a similar trend, with significantly higher values in *O. patens* individuals both with (2.0 ± 1.0‰) and without (1.3 ± 1.3‰) coralloid rhizomes compared to autotrophic references (−1.8 ± 1.3‰; 1.0 × 10^−7^ for both groups). However, the difference in δ^15^N values between *O. patens* with and without coralloid rhizomes was not statistically significant (*P* = 0.27). Notably, the δ^15^N values for *O. patens* protocorms (−0.7 ± 0.7‰, *n* = 5) were significantly lower than those of *O. patens* samples with coralloid rhizomes (1.9 ± 1.1‰, *n* = 15, *P* = 2.56 × 10^−4^) and *O. patens* samples without coralloid rhizomes (1.2 ± 1.1‰, *n* = 18, *P* = 0.01). Since these protocorms contain both fungal and plant tissues, their lower ^15^N enrichment (compared to adult leaves) may be attributed to the incorporation of fungal biomass, as observed in other mycoheterotrophic orchids (Suetsugu et al., 2024).

The ^13^C and ^15^N enrichment factors in *O. patens* with coralloid rhizomes were 3.9 ± 1.7‰ and 3.8 ± 1.1‰, respectively, while these values in those without coralloid rhizomes were 1.3 ± 1.7‰ and 3.1 ± 1.5‰, respectively. An isotope mixing model, assuming a linear relationship between carbon assimilation from fungal sources and ^13^C enrichment, estimated that approximately 58.2 ± 24.8% of carbon in *O. patens* with coralloid rhizomes is derived from fungal sources. In contrast, it was estimated that individuals without rhizomes obtain about 19.0 ± 25.8% of their carbon from fungi.

## DISCUSSION

The adaptive significance of mycoheterotrophic nutrition in mixotrophic plants remains insufficiently explored. Here, we demonstrated that *O. patens* with coralloid rhizomes predominantly associates with saprotrophic Psathyrellaceae fungi and exhibits significant ^13^C enrichment, indicating a greater reliance on fungal‐derived nutrients. Our observations further reveal that individuals more dependent on fungal‐derived nutrients produce more vigorous flowering scapes and a higher number of flowers. Since coralloid rhizome‐bearing plants do not exhibit reduced chlorophyll fluorescence and, although not statistically significant, tend to have larger leaf areas, the increased proportion of heterotrophic carbon in *O. patens* with coralloid rhizomes is unlikely to result from reduced photosynthesis. Instead, it likely reflects an actual increase in the carbon budget. Consequently, we conclude that coralloid rhizomes and the associated increase in mycoheterotrophy confer an advantage to *O. patens*.

Intriguingly, the closely related, leafless genus *Corallorhiza* predominantly relies on ECM fungi like Russulaceae or Thelephoraceae (Barrett et al., 2010, 2022; Taylor et al., 2004; Taylor & Bruns, 1997, 1999; Zimmer et al., 2008). Given that *O. indica* and all examined *Corallorhiza* species exploit ECM fungi, it is likely that ECM associations represent the ancestral condition among *Corallorhiza* species. Thus, the shift from rhizoctonia fungi to Psathyrellaceae in *O. patens* might have occurred independently, distinct from the shift from rhizoctonias to ECM fungi in *Corallorhiza*. An alternative explanation is a transition from ECM fungi to Psathyrellaceae, but since *O. patens* without coralloid rhizomes primarily associates with rhizoctonias, the former scenario seems more plausible. A more comprehensive understanding of shifts in mycorrhizal associations could be gained by analyzing mycorrhizal data from additional *Oreorchis* species and elucidating the precise phylogenetic relationships within the *Oreorchis–Corallorhiza* clade.

The divergence in fungal symbiosis – *O. patens* associating with saprotrophic non‐rhizoctonia fungi versus *O. indica* and *Corallorhiza* species associating with ECM fungi – may be linked to differing climatic conditions. *O. patens* thrives in warmer, moist forest environments (Maekawa, 1971), where saprotrophic fungi are likely more active in decomposing organic matter. In contrast, *O. indica* and *Corallorhiza* species are found in cooler, subboreal regions (Maekawa, 1971; Suetsugu, Haraguchi, et al., 2021). In temperate areas, highly mycoheterotrophic orchids typically form symbiotic relationships with ECM fungi, facilitating nutrient transfer from trees to orchids (Bidartondo et al., 2004; Hynson et al., 2013; Jacquemyn & Merckx, 2019; Motomura et al., 2010). Conversely, in warmer and more humid regions, fully mycoheterotrophic orchids often recruit saprotrophic non‐rhizoctonia fungi as their mycobionts (Hynson et al., 2013; Lee et al., 2015; Martos et al., 2009; Ogura‐Tsujita et al., 2009; Suetsugu, Matsubayashi, & Tayasu, 2020). For example, the fully mycoheterotrophic genus *Epipogium* includes the subtropical *E. roseum*, which associates with saprotrophic Psathyrellaceae (Yagame et al., 2008; Yamato et al., 2005), and the cool‐temperate *E. aphyllum*, which relies on ECM fungi, predominantly *Inocybe* spp. (Liebel & Gebauer, 2011; Minasiewicz et al., 2022; Roy et al., 2009).

Our study indicates that the subterranean morphology of *O. patens* significantly impacts both its fungal associations and nutritional mode. Plants without coralloid rhizomes favor associations with rhizoctonias and exhibit reduced heterotrophic carbon gain. In contrast, Psathyrellaceae taxa were particularly dominant in coralloid rhizomes of coralloid rhizome‐bearing individuals, although the major mycorrhizal symbionts in the roots of coralloid rhizome‐bearing plants remain Psathyrellaceae despite a slight increase in rhizoctonia associations. This observation is consistent with findings in *Cremastra appendiculata*, other members of the Calypsoinae, where individuals with coralloid rhizomes exclusively associate with Psathyrellaceae fungi, obtaining more than half of their carbon from these wood‐decaying fungi (Suetsugu et al., 2022; Yagame et al., 2021; Zahn et al., 2022). In contrast, mature *C. appendiculata* lacking coralloid rhizomes and interacting with rhizoctonias are likely fully autotrophic, based on ^2^H, ^13^C, and ^15^N isotopic data (Yagame et al., 2021; Zahn et al., 2022).

The estimation of carbon gain in *O. patens* plants lacking coralloid rhizomes based on ^13^C abundance may be underestimated, as rhizoctonias exhibit relatively low ^13^C enrichment (Gomes et al., 2023; Zahn et al., 2023). However, despite some associations with Psathyrellaceae fungi (and occasionally ECM fungi in the Ekari population), *O. patens* plants without coralloid rhizomes at the Ebetsu and Ekari populations exhibit significantly lower ^13^C enrichment than those with coralloid rhizomes. Thus, the variation in ^13^C signatures observed in *O. patens* – with or without coralloid rhizomes – likely reflects actual shifts in nutritional mode.

Given that coralloid rhizomes might be ontogenetically persistent protocorms retaining immature morphology (Freudenstein, 1994; Maekawa, 1971), and all orchids depend on fungi for their carbon demand at the protocorm stage (Merckx, 2013), it is reasonable that plants with coralloid rhizomes show higher levels of heterotrophy. If coralloid organs are persistent protocorms, the mycorrhizal communities in adult coralloid rhizomes likely mirror those supporting protocorms, a hypothesis warranting further study. Notably, coralloid rhizomes were reported over a century ago in the Calypsoinae orchid *Aplectrum hymenale* near decaying wood (Gillman, 1876; MacDougal, 1899), with early studies suggesting they facilitate fungal parasitism. Our findings in *O. patens* support this hypothesis, linking coralloid rhizomes to pronounced mycoheterotrophy and potential fitness advantages. This suggests that variable mycoheterotrophy via coralloid rhizomes may be a common adaptation among Calypsoinae green orchids on woody substrates.

**Figure 6:**
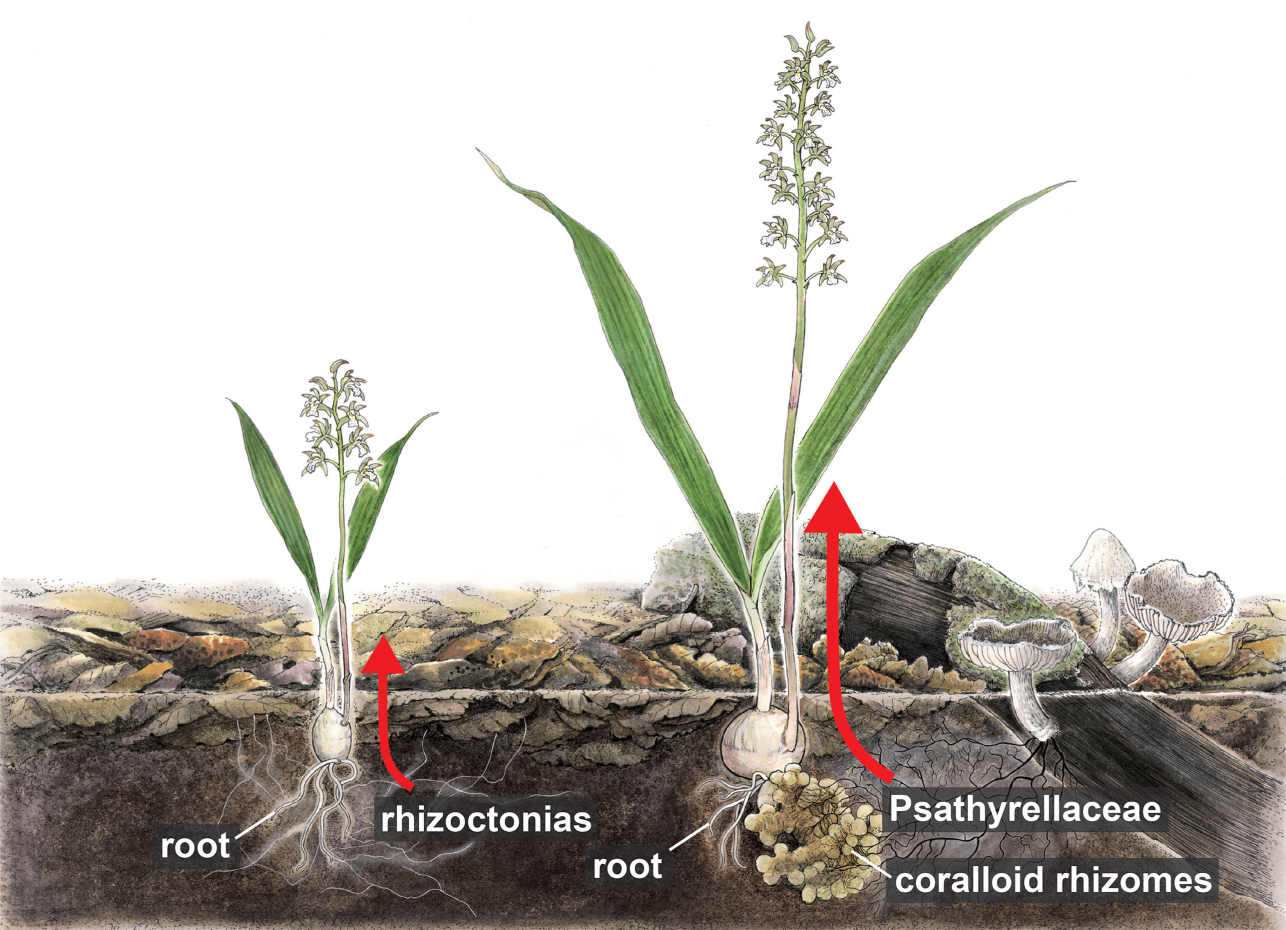
Proposed interactions between *Oreorchis patens* and its associated fungi. *Oreorchis* plants with coralloid rhizomes (right) show greater dependence on mycoheterotrophy by associating with wood‐decaying Psathyrellaceae fungi rather than rhizoctonias, compared to plants without coralloid rhizomes (left). Accessing carbon from decomposing wood allows these plants to utilize substantial carbon reservoirs, leading to more vigorous flowering scapes and a higher number of flowers. The length of the arrows represents the amount of carbon transfer from fungi to the plant.

The ability to exploit carbon from decomposing wood provides advantages by accessing substantial carbon reservoirs (Suetsugu et al., 2022). Since carbon availability can limit many organisms, including autotrophic species under low‐light conditions (Selosse & Roy, 2009), the acquisition of substantial heterotrophic carbon likely supports the growth of *O. patens* with coralloid rhizomes. Notably, flowering *O. patens* plants with coralloid rhizomes often exhibit a more robust stature, with taller flowering scapes and a higher number of flowers compared to those without coralloid rhizomes (Figure 6). This suggests that the presence of coralloid rhizomes and increased mycoheterotrophy positively impact plant vigor, highlighting the adaptive significance of mycoheterotrophy.

It is noteworthy that coralloid rhizomes in *O. patens* are rarely found during the mature stages of growth, with less than 10% of flowering individuals observed to have them, despite their likely beneficial impact. Similarly, Zahn et al. (2022) reported that adult *C. appendiculata* did not display coralloid rhizomes, while seedlings with very young leaves possessed both coralloid rhizomes and roots. However, as these seedlings approached maturity, they detached from the rhizomes (Zahn et al., 2022). A possible explanation is that saprotrophic Psathyrellaceae fungi – abundant in decomposed wood – are probably ephemeral; after compatible wood resources vanish, the plant likely relies on rhizoctonias as a fallback, albeit with lower vigor. Future research is needed to understand why *O. patens* and some other Calypsoinae taxa tend to associate more with rhizoctonias, which support only a low level of mycoheterotrophy at the adult stage, rather than with wood‐decaying fungi that likely ensure a higher degree of mycoheterotrophy (McCormick et al., 2021; Taylor & McCormick, 2008; Zahn et al., 2022).

The observed pattern in *O. patens* and some Calypsoinae taxa is likely related to the presence and abundance of different fungal types in the soil (McCormick et al., 2009; McCormick & Jacquemyn, 2014). While Psathyrellaceae fungi probably serve as the most effective mycorrhizal partners, they might not always be accessible, leading to the use of suboptimal mycorrhizal partners like rhizoctonias. Notably, mature *O. patens* with coralloid rhizomes have been found solely near decomposed fallen trees, indicating specific habitat requirements. Moreover, Psathyrellaceae fungi often emerge in the later stages of fungal succession on decaying wood (Ottosson et al., 2014; Rajala et al., 2012), potentially limiting the persistence of mature individuals with coralloid rhizomes.

In conclusion, our study showed that the alliance of *O. patens* with saprotrophic non‐rhizoctonia fungi diverges from the strategies of closely related species that typically associate with ECM fungi. Plants with coralloid rhizomes and high mycoheterotrophy exhibit more robust flowering scapes and increased flower numbers, suggesting that mycoheterotrophy substantially enhances plant vigor. Our findings provide insight into the adaptive significance of mycoheterotrophy in plants. Finally, the phenotypic plasticity in fungal association, underground morphology, and nutritional mode in *O. patens* likely opens new avenues for investigating the mechanisms regulating the degree of mycoheterotrophy.

## MATERIALS AND METHODS

### Field study

Fieldwork was conducted in three temperate forests: Ebetsu and Jozankei (both in Hokkaido Prefecture) and Ekari (Iwate Prefecture) from June 2010 to November 2021 (Table S1). Each site hosted over 100 mature *Oreorchis patens* specimens during the observation period. Most plants were located near decomposing logs on the forest floor or beneath the litter layer. Although flowering *O. patens* rarely developed coralloid rhizomes (less than 10% in our observations), our research prioritized those with coralloid rhizomes (Figure 1).

We collected mycorrhizal samples from 16 and 10 plants with and without coralloid rhizomes, respectively, in the Ebetsu population; 6 and 4 plants with and without coralloid rhizomes, respectively, in the Jozankei population; and 24 plants each with and without coralloid rhizomes, respectively, in the Ekari population for mycobiont molecular identification. To minimize disturbance, we carefully extracted minimal root or rhizome segments (2–4 fragments, approximately 2–3 cm each) by digging about 20 cm from the flowering scapes and approaching the roots or coralloid rhizomes laterally. To compare fungi associated with roots versus rhizomes in coralloid rhizome‐bearing plants, we separated these tissues during sampling. After sampling, we refilled the excavated holes with the original soil. During this process, we encountered small, coralloid protocorms (Figure 1) near mature plants in the Ekari population. These were likely *O. patens* protocorms, as coralloid protocorms are characteristic of Calypsoinae orchids (Freudenstein, 1994; Maekawa, 1971; Zahn et al., 2022), and no other Calypsoinae orchids with coralloid protocorms were found in the area. Due to their small size, these probable *O. patens* protocorms (hereafter referred to as *O. patens* protocorms) were not divided for mycorrhizal identification and stable isotope analysis. Instead, we prioritized stable isotope analysis to serve as a reference for a fully mycoheterotrophic endpoint.

For isotopic analysis, to minimize the influence of site‐specific factors affecting isotopic composition, we harvested leaves of at least three reference plants at the same height as the focal *O. patens* individuals within 2 m × 2 m quadrats, following Gebauer and Meyer (2003). At each location – Ebetsu, Jozankei, and Ekari – five 2 m × 2 m quadrats were established on June 30, 2010, October 28, 2018, and May 14, 2021, respectively, to study the physiological ecology of *O. patens*. The collected samples comprised four and eight plants with and without coralloid rhizomes from the Ebetsu population, four and five plants with and without coralloid rhizomes from the Jozankei population, and eight and six plants with and without coralloid rhizomes from the Ekari population.

Morphological and physiological parameters were recorded for the plants used in the isotopic analysis in the Ekari population, excluding one individual with coralloid rhizomes whose flowering scape was broken. The measurements included characteristics related to flowering scapes (scape height and number per individual), leaves (number of leaves per individual, as well as length and width of fully developed leaves), and the number of flowers. Chlorophyll fluorescence (*F*_v_/*F*_m_) of the leaves was measured using the FluorPen FP100 (Photon Systems Instruments, Brno, Czech Republic), following Shutoh et al. (2020) Suetsugu, Yamato, et al. (2021). To evaluate the influence of coralloid rhizomes on these traits, we employed a linear model with the “presence of coralloid rhizomes” as an explanatory variable. A *post hoc* Tukey–Kramer test was used to determine if there were significant differences between the two groups.

### Molecular identification of mycobionts

We collected mycorrhizal fragments (3 mm in length) from both root and rhizome tissues under a light microscope. Each sample was washed to remove small surface particles and then subjected to surface sterilization by submerging it in 1% sodium hypochlorite for 30 sec, followed by three 30‐sec rinses in sterile distilled water. After surface sterilization, DNA was extracted from the mycorrhizal samples using the cetyltrimethylammonium bromide method (Doyle & Doyle, 1990).

The ITS region sequences of mycorrhizal fungi were amplified using the ITS86F/ITS4 primer set, which has proven effective in studying fungal communities within orchid roots (Waud et al., 2014). These primers were fused with 3–6‐mer Ns and Illumina forward/reverse sequencing primers, following the protocol of Suetsugu, Haraguchi, et al. (2021). PCR amplification was conducted using the Q5 High‐Fidelity DNA Polymerase kit. The reaction conditions included an initial denaturation at 98°C for 40 sec, followed by 35 cycles of 98°C for 5 sec, 58°C for 10 sec, and 72°C for 20 sec, with a final extension at 72°C for 10 min. To incorporate Illumina P5/P7 adapter sequences and sample‐specific indices (Suetsugu & Matsubayashi, 2021a; Syed et al., 2009), a supplementary PCR was performed. The supplemental PCR conditions involved an initial denaturation at 98°C for 40 sec, followed by 12 cycles of 98°C for 5 sec, 65°C for 10 sec, and 72°C for 20 sec, with a final extension at 72°C for 10 min. The pooled library was subjected to sequencing using the Illumina MiSeq sequencer with the MiSeq Reagent Micro Kit v2 (300 cycles). The sequence data have been deposited in the NCBI Sequence Read Archive (SRA accession number PRJNA1213319).

After sequencing, we conducted a bioinformatic analysis using Claident v0.9.2024.06.10 (Tanabe & Toju, 2013), as described by Suetsugu and Okada (2021). Briefly, after removing primer regions and eliminating low‐quality reads, erroneous sequences were denoised using DADA2 (Callahan et al., 2016) implemented in Claident. Subsequently, we removed sequences that could have originated from PCR chimera formation and index hopping using the clremovechimev and clremovecontam commands in Claident (Esling et al., 2015; Nilsson et al., 2019). Finally, the remaining sequencing reads were clustered into OTUs with a threshold similarity of 97% using VSEARCH v2.8.0 (Rognes et al., 2016), which was implemented in the clclassseqv command. The most abundant sequence within each OTU cluster was designated as the representative sequence for further analysis. Taxonomic assignment of the OTUs was performed based on the query‐centric auto‐*k*‐nearest‐neighbor (QCauto) and the lowest common ancestor (LCA) algorithms (Huson et al., 2007), using the “overall_genus” reference database in Claident. Only those OTUs assigned to potentially orchid mycorrhizal fungi (Dearnaley et al., 2012; Wang et al., 2021) were retained for further analysis.

Since Psathyrellaceae and rhizoctonias were identified as the main mycobionts of *O. patens* with and without coralloid rhizomes, respectively (see “Results” section), we constructed the phylogenetic tree of the OTUs belonging to these families and their closely related fungi to further explore their relationships. These OTUs, identified as *O. patens* mycobionts, were subjected to a BLAST search against the International Nucleotide Sequence Database Collaboration (INSDC) for comparison (Altschul et al., 1997). Several phylogenetically close sequences, along with representative sequences from these families, were downloaded.

Multiple sequence alignments were performed using MAFFT v7.475 (Katoh & Standley, 2013) with the L‐INS‐i option for Psathyrellaceae (*Coprinopsis*, *Coprinellus*, *Psathyrella*) and Ceratobasidiaceae, while PRANK (Löytynoja, 2021) was employed for Sebacinales and Tulasnellaceae. Subsequently, maximum‐likelihood phylogenetic trees were constructed using IQ‐TREE 1.6.12 (Nguyen et al., 2015). The best‐fit substitution models were selected by ModelFinder (Kalyaanamoorthy et al., 2017) based on the Bayesian Information Criterion (BIC). To assess the reliability of the phylogenetic tree, branch support was calculated using two methods: the Shimodaira–Hasegawa‐like approximate likelihood ratio test (SH‐aLRT) (Guindon et al., 2010) and ultrafast bootstrapping (UFboot) (Minh et al., 2013), both with 1000 replicates. Nodes with SH‐aLRT values ≥80% and ultrafast bootstrap values ≥95% were considered to have strong support.

### δ^13^C and δ^15^N analysis

The natural abundance of ^13^C and ^15^N isotopes in the *O. patens* and co‐occurring autotrophic reference plants was quantified using a continuous‐flow isotope ratio mass spectrometer connected to an elemental analyzer (Thermo Fisher Scientific, Waltham, MA, USA), as described by Suetsugu and Matsubayashi (2021b). Relative isotope abundances were calculated and denoted as:

$$\delta^{13}C\;\text{or}\;\delta^{15}N=\left(R_{\text{sample}}/R_{\text{standard}}–1\right)\times 1000\;\left[‰\right],$$

where *R*_sample_ represents the ^13^C/^12^C or ^15^N/^14^N ratio of each sample, and *R*_standard_ represents the ^13^C/^12^C or ^15^N/^14^N ratios of Vienna PeeDee Belemnite or atmospheric N_2_, respectively. Calibration of the C and N isotope ratios was achieved using two laboratory standards: CERKU‐03 (glycine, δ^13^C = −34.92‰, δ^15^N = 2.18‰) and CERKU‐05 (threonine, δ^13^C = −9.45‰, δ^15^N = −2.88‰) (Tayasu et al., 2011). The analytical standard deviations (SDs) obtained from repeated measurements of these standards were less than 0.06‰ for δ^13^C (*n* = 54) and 0.11‰ for δ^15^N (*n* = 53).

To enable comparisons across sites, enrichment factors (ε^13^C and ε^15^N) were also determined as the differences between the *δ* values of each specimen and the mean δ values of neighboring autotrophic plants within the same plot (Preiss & Gebauer, 2008). To determine the proportion of carbon derived from fungi (% Cdf) in *O. patens* specimens with or without coralloid rhizomes, we applied a linear two‐source mixing model: % Cdf = (ε^13^Cpmh/ε^13^Cfmh) × 100. Here, ε^13^Cpmh corresponds to the ^13^C enrichment factor of an *O. patens* plant with or without coralloid rhizomes, and ε^13^Cfmh represents the mean ^13^C enrichment factor of *O. patens* at the protocorm stage. Finally, we employed a linear mixed model to compare the δ^13^C, δ^15^N, ε^13^C, and ε^15^N values among different groups, fitting ‘identity’ as a fixed term and ‘plot’ and ‘population’ as random terms. A post hoc Tukey–Kramer test was then utilized to determine whether the values among *O. patens* plants with coralloid rhizomes, *O. patens* plants without coralloid rhizomes, *O. patens* protocorms, and autotrophic reference plants differ significantly.

## AUTHOR CONTRIBUTIONS

KS planned and designed the research, conducted fieldwork and laboratory experiments, carried out analyses, and drafted the initial manuscript. HO performed analyses, revised the manuscript, and approved the final version for publication.

## CONFLICT OF INTEREST

We declare that we have no competing interests.

## Supporting information


**Figure S1.** Phylogenetic tree of ITS2 rDNA sequences from *Psathyrella* OTUs detected in mycorrhizal samples of *Oreorchis patens* (in bold), along with sequences obtained from the INSDC database.
**Figure S2.** Phylogenetic tree of ITS2 rDNA sequences from *Candolleomyces* OTUs detected in mycorrhizal samples of *Oreorchis patens* (in bold), along with sequences obtained from the INSDC database.
**Figure S3.** Phylogenetic tree of ITS2 rDNA sequences from *Coprinellus* OTUs detected in mycorrhizal samples of *Oreorchis patens* (in bold), along with sequences obtained from the INSDC database.
**Figure S4.** Phylogenetic tree of ITS2 rDNA sequences from Tulasnellaceae OTUs detected in mycorrhizal samples of *Oreorchis patens* (in bold), along with sequences obtained from the INSDC database.
**Figure S5.** Phylogenetic tree of ITS2 rDNA sequences from Sebacinales OTUs detected in mycorrhizal samples of *Oreorchis patens* (in bold), along with sequences obtained from the INSDC database.

**Table S1.** The sequencing reads of OTUs detected in each *Oreorchis patens* individual.
**Table S2.** Values of δ^13^C, δ^15^N, ε^13^C and ε^15^N for each sample of *Oreorchis patens* with and without coralloid rhizomes, *O. patens* protocorms, and the surrounding.

## Data Availability

The sequence data have been deposited in the NCBI Sequence Read Archive (SRA accession number PRJNA1213319).
