# Supplementary material for: Subterranean morphology underpins the degree of mycoheterotrophy, mycorrhizal associations, and plant vigor in a green orchid Oreorchis patens
Source: Plant J. 2025 Feb 19;121(4):e70045. doi: 10.1111/tpj.70045 (PMC11837900; doi:10.1111/tpj.70045)
Supplement: Supplementary file 1 — Figure S1. Phylogenetic tree of ITS2 rDNA sequences from Psathyrella OTUs detected in mycorrhizal samples of Oreorchis patens (in bold), along with sequences obtained from the INSDC database. Figure S2. Phylogenetic tree of ITS2 rDNA sequences from Candolleomyces OTUs detected in mycorrhizal samples of Oreorchis patens (in bold), along with sequences obtained from the INSDC database. Figure S3. Phylogenetic tree of ITS2 rDNA sequences from Coprinellus OTUs detected in mycorrhizal samples of Oreorchis patens (in bold), along with sequences obtained from the INSDC database. Figure S4. Phylogenetic tree of ITS2 rDNA sequences from Tulasnellaceae OTUs detected in mycorrhizal samples of Oreorchis patens (in bold), along with sequences obtained from the INSDC database. Figure S5. Phylogenetic tree of ITS2 rDNA sequences from Sebacinales OTUs detected in mycorrhizal samples of Oreorchis patens (in bold), along with sequences obtained from the INSDC database. [file TPJ-121-0-s001.docx]

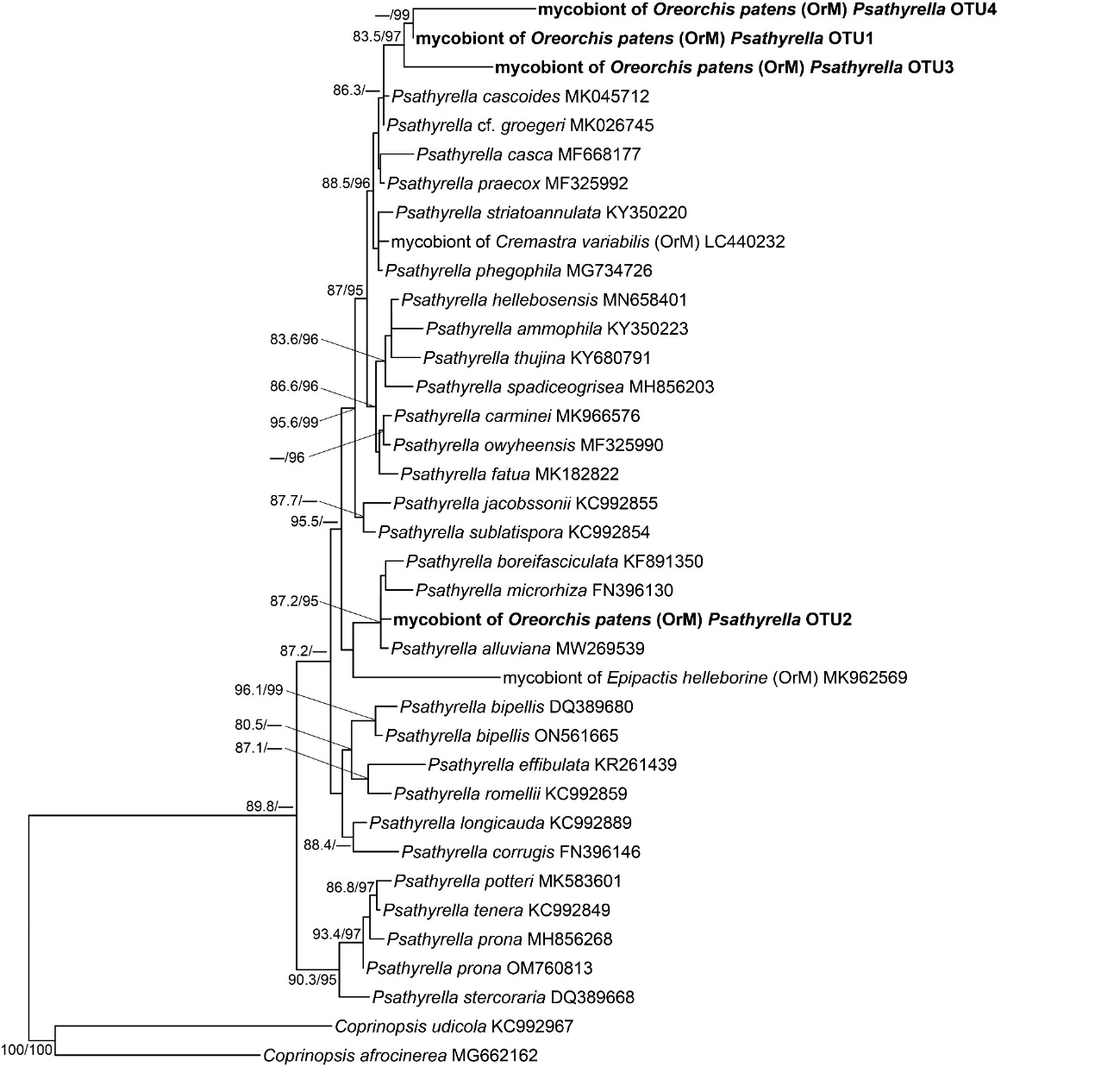


**Figure S1.** Phylogenetic tree of ITS2 rDNA sequences from *Psathyrella* OTUs detected in mycorrhizal samples of *Oreorchis patens* (in bold), along with sequences obtained from the INSDC database. The OTUs detected in *Oreorchis patens* are ranked by the number of sequencing reads. Accession numbers are provided for all INSDC sequences. The tree is rooted using *Coprinopsis udicola* and *Coprinopsis afrocinerea* (Psathyrellaceae). Nodes with SH-aLRT values < 80% and ultrafast bootstrap values < 95% are not shown. Scale bar indicates the number of substitutions per site. OrM: Orchid mycorrhizal fungi.


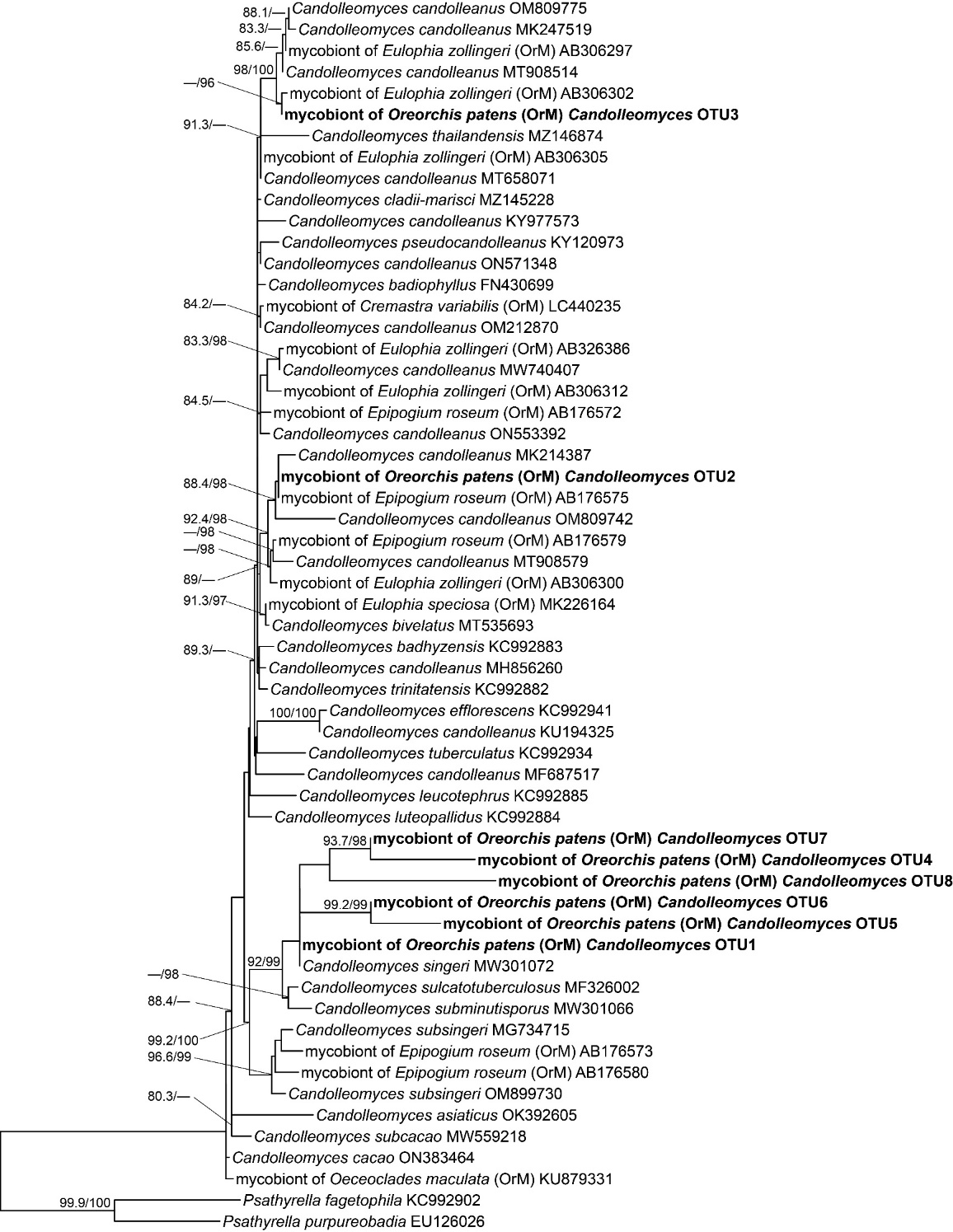


**Figure S2.** Phylogenetic tree of ITS2 rDNA sequences from *Candolleomyces* OTUs detected in mycorrhizal samples of *Oreorchis patens* (in bold), along with sequences obtained from the INSDC database. The OTUs detected in *Oreorchis patens* are ranked by the number of sequencing reads. Accession numbers are provided for all INSDC sequences. The tree is rooted using *Psathyrella fagetophila* and *Psathyrella purpureobadia* (Psathyrellaceae). Nodes with SH-aLRT values < 80% and ultrafast bootstrap values < 95% are not shown. Scale bar indicates the number of substitutions per site. OrM: Orchid mycorrhizal fungi.


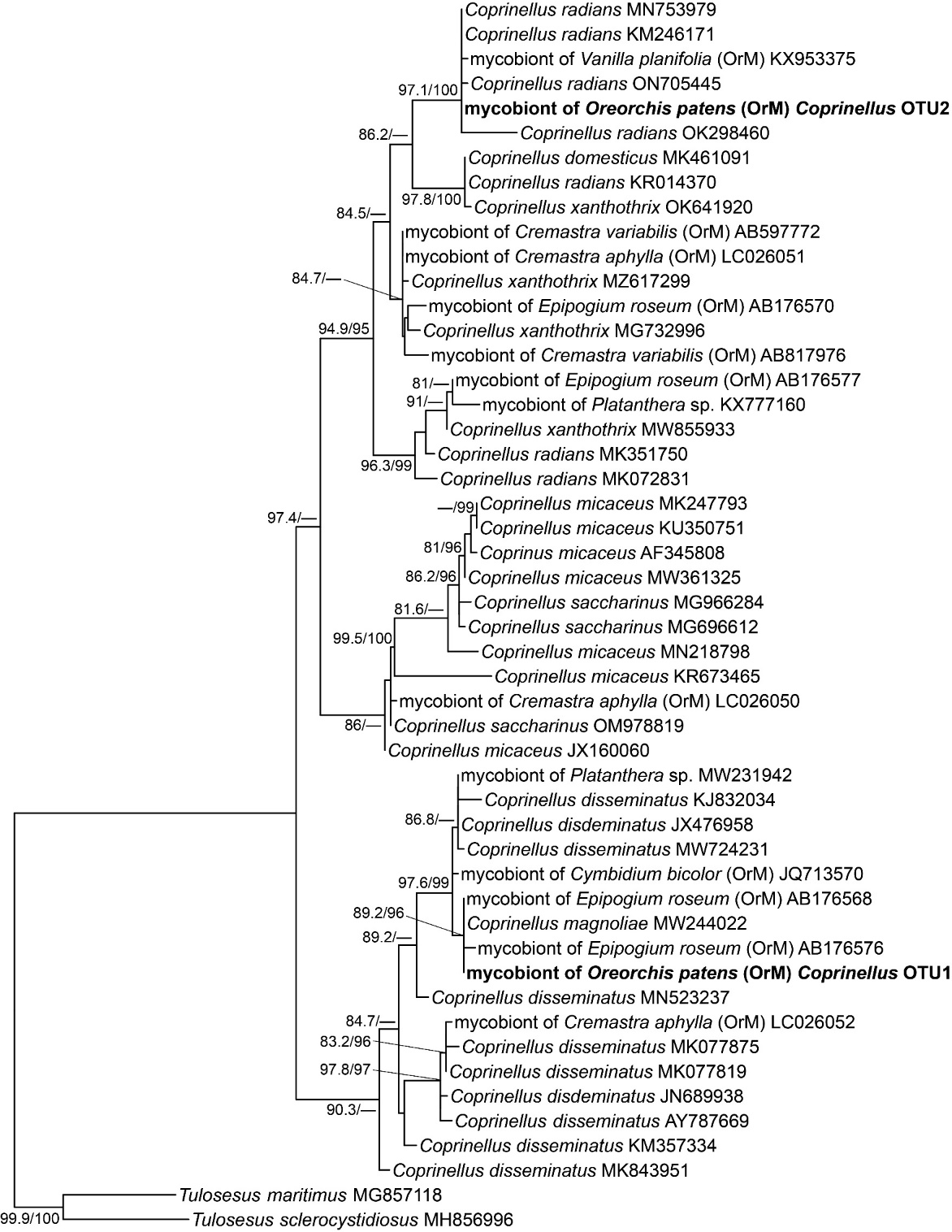


**Figure S3.** Phylogenetic tree of ITS2 rDNA sequences from *Coprinellus* OTUs detected in mycorrhizal samples of *Oreorchis patens* (in bold), along with sequences obtained from the INSDC database. Accession numbers are provided for all INSDC sequences. The OTUs detected in *Oreorchis patens* are ranked by the number of sequencing reads. The tree is rooted using *Tulosesus maritimus* and *Tulosesus sclerocystidiosus* (Psathyrellaceae). Nodes with SH-aLRT values < 80% and ultrafast bootstrap values < 95% are not shown. Scale bar indicates the number of substitutions per site. OrM: Orchid mycorrhizal fungi.


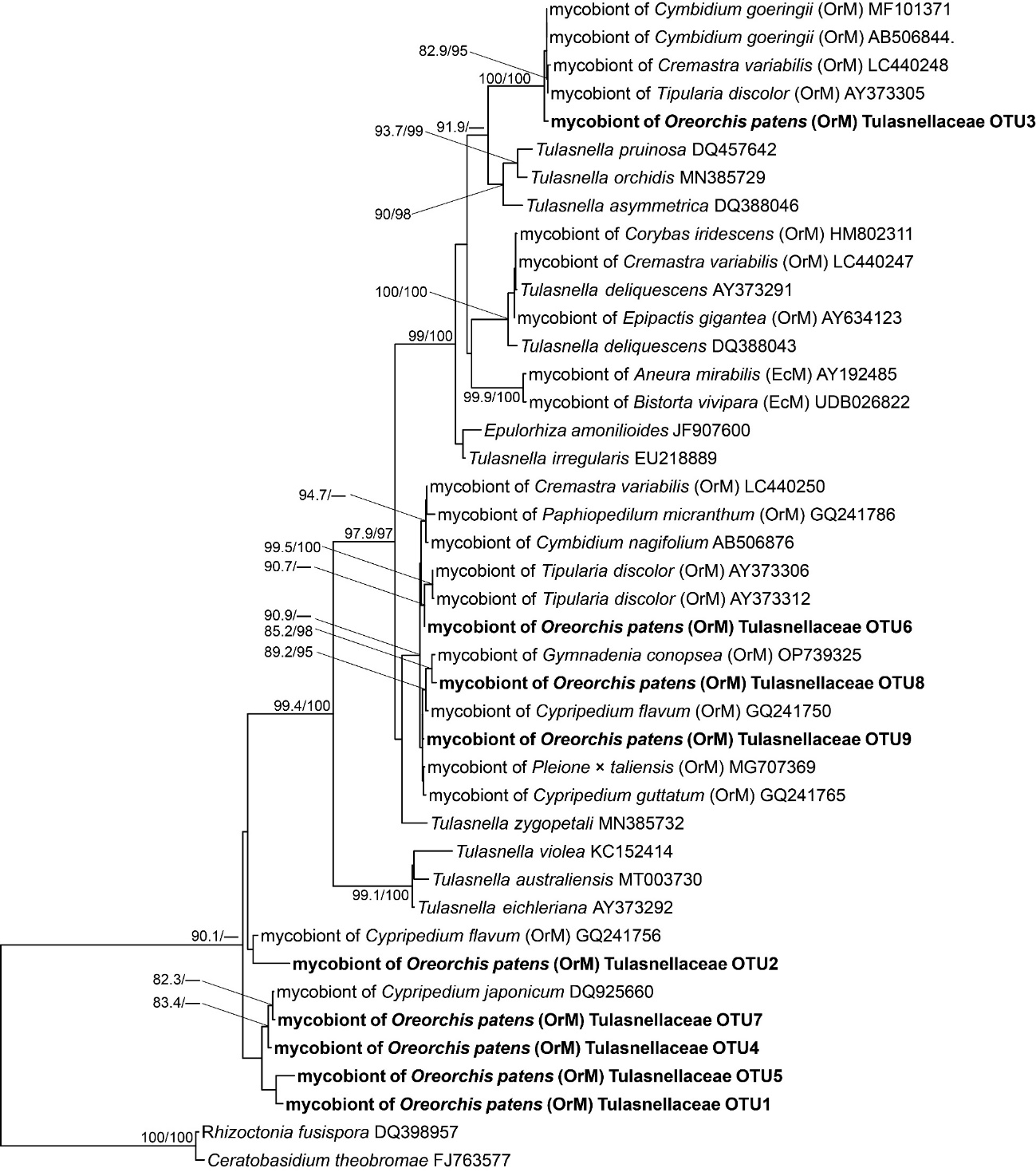


**Figure S4.** Phylogenetic tree of ITS2 rDNA sequences from Tulasnellaceae OTUs detected in mycorrhizal samples of *Oreorchis patens* (in bold), along with sequences obtained from the INSDC database. The OTUs detected in *Oreorchis patens* are ranked by the number of sequencing reads. Accession numbers are provided for all INSDC sequences. The tree is rooted using *Rhizoctonia fusispora* and *Ceratobasidium theobromae* (Ceratobasidiaceae). Nodes with SH-aLRT values < 80% and ultrafast bootstrap values < 95% are not shown. Scale bar indicates the number of substitutions per site. EcM: Ectomycorrhizal fungi. OrM: Orchid mycorrhizal fungi.


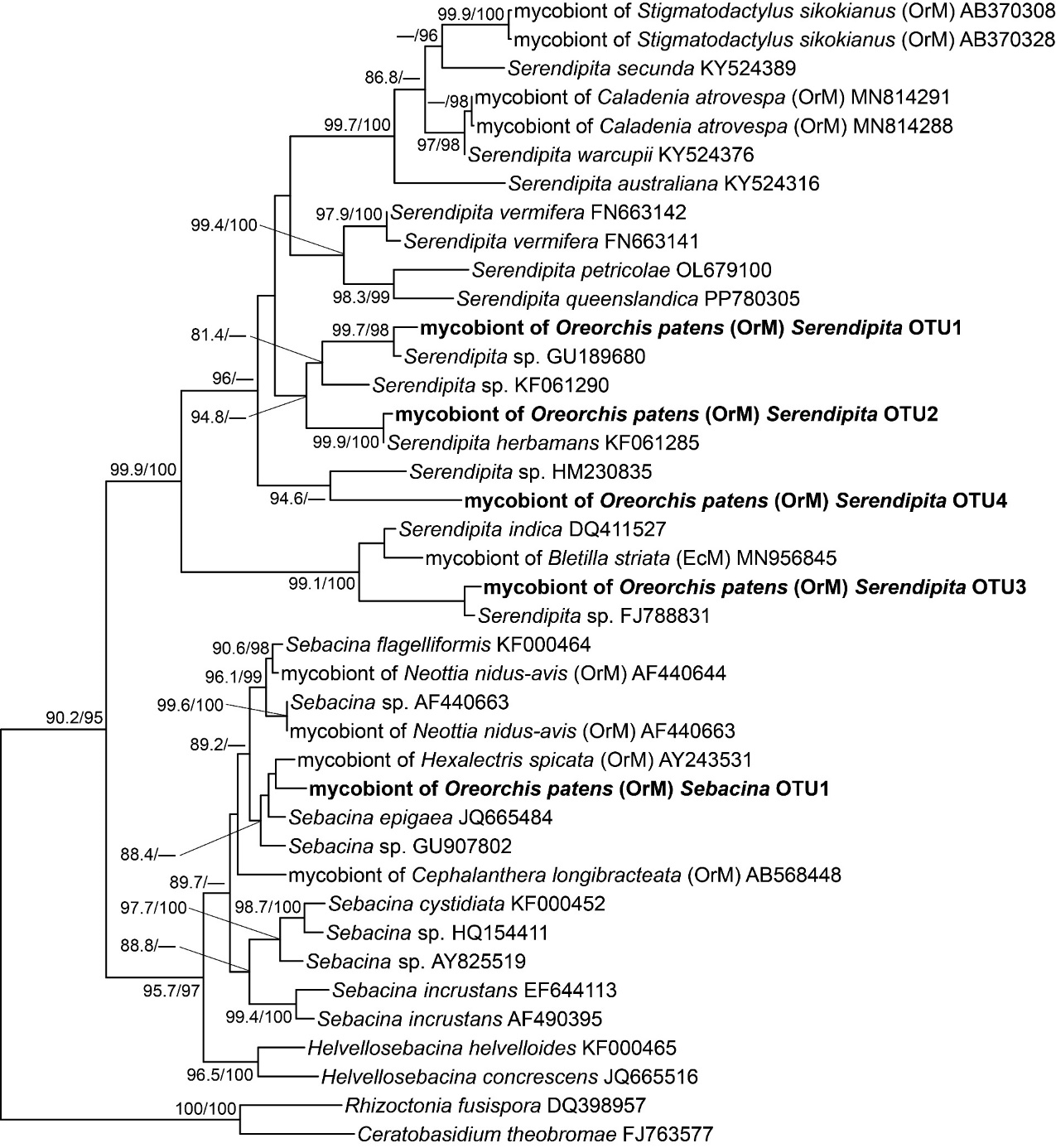


**Figure S5.** Phylogenetic tree of ITS2 rDNA sequences from Sebacinales OTUs detected in mycorrhizal samples of *Oreorchis patens* (in bold), along with sequences obtained from the INSDC database. The OTUs detected in *Oreorchis patens* are ranked by the number of sequencing reads. Accession numbers are provided for all INSDC sequences. The tree is rooted using *Rhizoctonia fusispora* and *Ceratobasidium theobromae* (Ceratobasidiaceae). Nodes with SH-aLRT values < 80% and ultrafast bootstrap values < 95% are not shown. Scale bar indicates the number of substitutions per site. OrM: Orchid mycorrhizal fungi.
